# MicroRNAs, Long Non-Coding RNAs, and Circular RNAs: Potential Biomarkers and Therapeutic Targets in Pheochromocytoma/Paraganglioma

**DOI:** 10.3390/cancers13071522

**Published:** 2021-03-26

**Authors:** Peter Istvan Turai, Gábor Nyírő, Henriett Butz, Attila Patócs, Peter Igaz

**Affiliations:** 1 Department of Endocrinology, Department of Internal Medicine and Oncology, Faculty of Medicine, Semmelweis University, Korányi str. 2/a, H-1083 Budapest, Hungary; peteturai@gmail.com; 2 MTA-SE Molecular Medicine Research Group, H-1083 Budapest, Hungary; nyirogabor1@gmail.com; 3 Department of Laboratory Medicine, Faculty of Medicine, Semmelweis University, H-1089 Budapest, Hungary; butz.henriett@med.semmelweis-univ.hu (H.B.); patocs.attila@med.semmelweis-univ.hu (A.P.); 4 Department of Molecular Genetics, National Institute of Oncology, H-1122 Budapest, Hungary; 5 MTA-SE Hereditary Endocrine Tumors Research Group, H-1089 Budapest, Hungary

**Keywords:** pheochromocytoma, paraganglioma, genetics, non-coding RNA, malignancy, biomarker, treatment

## Abstract

**Simple Summary:**

Pheochromocytomas/paragangliomas (PPGL) are rare tumors originating from chromaffin tissues. Around 40% of pheochromocytomas/paragangliomas (PPGL) harbor germline mutations, representing the highest heritability among human tumors. Unfortunately, there are no available molecular markers for the metastatic potential of these tumors, and the prognosis of metastatic forms is rather dismal. In this review, we present the potential relevance of non-coding RNA molecules including microRNAs, long non-coding RNAs and circular RNAs in PPGL pathogenesis and diagnosis. The pathomechanisms presented might also represent potential novel therapeutic targets.

**Abstract:**

Around 40% of pheochromocytomas/paragangliomas (PPGL) harbor germline mutations, representing the highest heritability among human tumors. All PPGL have metastatic potential, but metastatic PPGL is overall rare. There is no available molecular marker for the metastatic potential of these tumors, and the diagnosis of metastatic PPGL can only be established if metastases are found at “extra-chromaffin” sites. In the era of precision medicine with individually targeted therapies and advanced care of patients, the treatment options for metastatic pheochromocytoma/paraganglioma are still limited. With this review we would like to nurture the idea of the quest for non-coding ribonucleic acids as an area to be further investigated in tumor biology. Non-coding RNA molecules encompassing microRNAs, long non-coding RNAs, and circular RNAs have been implicated in the pathogenesis of various tumors, and were also proposed as valuable diagnostic, prognostic factors, and even potential treatment targets. Given the fact that the pathogenesis of tumors including pheochromocytomas/paragangliomas is linked to epigenetic dysregulation, it is reasonable to conduct studies related to their epigenetic expression profiles and in this brief review we present a synopsis of currently available findings on the relevance of these molecules in these tumors highlighting their diagnostic potential.

## 1. Introduction

Non-coding RNA molecules encompassing microRNAs, long non-coding RNAs, and circular RNAs have been implicated in the pathogenesis of various tumors, and were also proposed as valuable diagnostic and prognostic factors, and even potential therapeutic targets. Given the fact that the pathogenesis of tumors including pheochromocytomas/paragangliomas (PPGL) is partly linked to epigenetic dysregulation [1], it is reasonable to investigate their epigenetic expression profiles. 

Pheochromocytomas are rare (incidence is approximately 0.8 per 100,000 people per year) catecholamine-producing endocrine tumors, arising from neural-crest-derived chromaffin cells. They have a strong genetic background and originate either in the adrenal medulla (80%) or in the sympathetic or parasympathetic paraganglia (20%), “extra-adrenal pheochromocytomas” (paraganglioma) as formerly referred to in [2]. A considerable proportion (40%) of pheocromocytoma/paraganglioma (PPGL) is diagnosed as manifestations of hereditary tumor syndromes, including familial paraganglioma syndrome types 1–5 (mutations in genes coding for subunits and associated factors of succinate dehydrogenase (*SDH*), e.g., *SDHB*, *SDHC*, *SDHD*, *SDHA* and *SDHAF2* (collectively called *SDHx*), von Hippel-Lindau syndrome (mutations of *VHL* tumor suppressor), multiple endocrine neoplasia type 2 (mutations of the *RET* protooncogene), neurofibromatosis type 1 (mutations of *NF1* tumor suppressor) and other germline mutations of various genes linked to major pathogenic processes in PPGL pathogenesis (e.g., *HIF2A*, *MAX*, *MDH2*, *FH*, *TMEM127*, *KIF1B*, *PHD/EGLN1*) [3,4,5]. At present, there are more than 12 genetic syndromes and 22 PPGL driver genes that contribute to PPGL formation [6,7]. This proportion of germline mutations has the highest degree of heritability among human tumors [8]. Moreover, sporadic PPGL were found to harbor somatic mutations in genes corresponding to their germline counterparts [9].

The molecular etiology of PPGL is especially important to explore as PPGL display various driver mutations with serious impact on diagnosis, prognosis and therapy as well. As a familial disease, early genetic diagnosis can not only facilitate the treatment of the proband, but is also an important step to detect potentially mutation carriers in the family [10]. Another reason for genetic testing is the well-known causative link between some driver mutations and their metastatic potential [8]. The rate of metastatic forms of catecholamine-secreting tumors is rather variable in different studies ranging between 5–26%. On the other side up to 50% of patients with metastatic PPGL have specific germline mutations [11,12,13]. The risk of metastasis is particularly high in individuals harboring germline *SDHB* mutations [12]. PPGL susceptibility can be associated with mutations either in tumor suppressor genes (e.g., *VHL*, *NF1*, *SDHB*) or in proto-oncogenes (e.g., *RET*, *HRAS*) [7]. 

**Figure 1:**
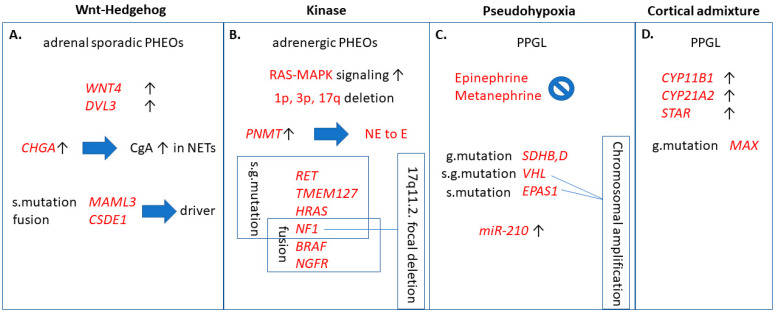
Clinically relevant functions of the four molecular pathways contributing to pheochromocytomas/paragangliomas (PPGL). (**A**) Wnt-Hedgehog overexpressed subtype included mainly adrenal sporadic pheochromocytomas and high chromogranin A levels. *MAML3* and *CSDE1* are independently important driver mutations leading to Wnt-Hedgehog activation. (**B**) Kinase signaling pathway is correlated to pheochromocytomas of adrenergic phenotype due to overexpression of *PNMT*, comprising somatic- and germline mutations and chromosomal deletions, as well. (**C**): Pseudohypoxia subtype, in addition to somatic- and germline mutations and chromosomal amplification, also exhibited overexpression of *miR-210*. (**D**) Overexpression of *CYP11B1*, *CYP21A2*, and *STAR* adrenal cortex markers was characteristic to cortical admixture subtype, along with *MAX* mutation in PPGL. g. mutation: germline mutation; s. mutation: somatic mutation; s.g. mutation: somatic and germline mutation; *WNT4*: wingless-related integration site 4; *DVL3*: dishevelled 3; *CHGA*: encodes chromogranin A (CgA); NET: neuroendocrine tumor; *MAML3*: mastermind-like transcriptional coactivator 3; *CSDE1*: cold shock domain containing E1; *RAS*: rat entry sarcoma; *MAPK*: mitogen-activated protein kinase; *PNMT*: phenylethanolamine N-methyltransferase; NE: norepineprhrine; E: epinephrine; *RET*: rearranged during transfection; *TMEM127*: transmembrane protein 127; HRAS: Harvey rat sarcoma viral oncogene homolog; *NF1*: neurofibromatosis 1; BRAF: v-raf murine sarcoma viral oncogene homolog B1; *NGFR*: nerve growth factor receptor; *SDH*: succinate dehydrogenase; *VHL*: Von-Hippel Lindau; *EPAS1*: endothelial PAS domain 1; *CYP11B1*: cytochrome P450 family 11 subfamily B member 1; *CYP21A2*: cytochrome P450 family 21 subfamily A member 2; *STAR*: steroid acute regulatory protein; *MAX*: myc associated factor X.

In order to further specify PPGL types and their tumor behavior, according to another recent paper, PPGL can further be classified into four molecular subtypes [14] (Figure 1). These groups include Wnt-altered, kinase signaling, pseudohypoxia, and cortical admixture subtypes with different molecular features and also clinical behavior. For example, the Wnt-altered subtype seems to be specific for sporadic PPGL as no germline mutations were observed within these tumors. The pseudohypoxia type generally had no epinephrine or metanephrine secretion, and also showed overexpression of the previously described tumor hypoxia marker microRNA-210 (*miR-210*) [15]. The cortical admixture type was found to be correlated with *MAX* (*MYC* associated factor X) mutations, which is also included as one of the susceptibility genes for hereditary PPGL [16]. Finally, kinase signaling exhibited the highest expression of *PNMT* (phenylethanolamine N-methyltransferase), an enzyme known to convert norepinephrine to epinephrine and according to that, was found mainly in pheochromocytomas.

From the clinical point of view, primary symptoms of excessive catecholamine secretion are episodic headache, sweating, and tachycardia (palpitations), also called the “classic triad” [17,18]. Either sustained or paroxysmal hypertension and even unexplained orthostatic hypotension are also characteristic features of PPGL. Other non-specific signs related to catecholamine-excess are anxiety, panic attacks, tremor, pallor, frequent urination, constipation, vision disturbances, hyperglycemia, and severe cardiovascular complications including stroke, aortic dissection, and stress-induced (takotsubo) cardiomyopathy [19]. In the so-called “pheochromocytoma crisis” patients suffer from hyperthermia, mental status change, and multisystem dysfunction, hence they require immediate medical attention [20]. Signs related to the general properties of a tumor are pain—depending on tumor location—weight loss, hematuria, and rarely erythrocytosis due to overproduction of erythropoietin [21]. Ever-increasingly, PPGL often appear with no associated symptoms as an incidental finding on imaging performed for other purposes (approximately 5–8% of adrenal incidentalomas), and also due to genetic screening in the context of familial disease [5]. 

Diagnosis of PPGL is based on a thorough clinical examination and medical history followed by biochemical tests, diagnostic imaging, and genetic testing. Biochemical tests include measuring 24 h urinary fractionated metanephrines and catecholamines or plasma metanephrine [22,23,24]. The general neuroendocrine tumor marker chromogranin A (CgA) is also useful. However, CgA is not specific for PPGL, but as its serum levels correlate with tumor burden, it is applicable for monitoring PPGL patients [25]. Patients with positive biochemical test results need to proceed on radiological evaluation, such as ^123^I-MIBG scan (meta-iodobenzylguanidine), MRI (magnetic resonance imaging), CT (computed tomography), ^18^FDG PET-CT (fluorodeoxyglucose positron emission tomography), or ^68^Ga-DOTATATE-PET (dodecanetetraacetic tyrosine-3-octreotate) [26]. 

Beside the clinical evaluation, at present, there are no reliable histomorphological features to distinguish between benign and metastatic PPGL, however Pheochromocytoma of the Adrenal Gland Scaled Score (PASS) and the Grading System for Adrenal Pheochromocytoma and Paraganglioma (GAPP) have been evaluated in a recent meta-analysis as promising tools with a good negative predictive value [27]. The recent WHO classification omitted the terms benign and malignant pheochromocytoma, and defined metastatic PPGL as a tumor with metastases at “extra-chromaffin” sites [28]. Patients with metastatic PPGL have poor prognosis with an estimated 44% overall survival (OS) at 5 years due to limited treatment options [29]. Whereas some patients present with synchronous metastases, metastases occur in several patients after the removal of the primary tumor, i.e., in a metachronous fashion. Long-term monitoring in all patients is warranted, even in those patients seemingly cured from the disease, which is obviously a life-long burden for such patients [30]. Metastasis in PPGL can occur as long as 53 years after surgery [31]. Unfortunately, despite intensive efforts, there are no reliable molecular markers of the metastatic potential of PPGL either. Altogether, according to the current WHO classification, all PPGL should be regarded as potentially malignant/metastatic, and followed up, but only a minority of PPGL will actually metastasize [32,33]. 

Currently, the primary treatment of PPGL is surgical resection, although removal of the tumor does not always lead to the cure of PPGL or to normotension [30]. However, it is possible that successful surgical treatment can not only be curative, but can also lead to normotension, normalization of blood pressure variability, and even normalization of urinary metanephrines [34]. Undiagnosed or not properly treated PPGL has high morbidity and mortality rate mainly due to cardiovascular complications. Other complications can also be life-threatening, such as drug interactions, hypertensive crises due to diagnostic- or therapeutic manipulations—owing to the sympathetic activation, and also malignancy or associated neoplasms [35]. For metastatic PPGL, there is no curative treatment, and currently available systemic chemotherapeutic approaches (e.g., CVD—cyclophosphamide-vincristin-dacarbazin chemotherapy) have limited efficacy [36]. Novel treatment options including *VEGF* (vascular endothelial growth factor) and tyrosine kinase inhibitors (e.g., axitinib, dovitinib, lenvatinib, sunitinib) exist for patients with *SDHA*, *SDHB*, *SDHD*, *RET*, *VHL*, and *FH* mutations in renal cell carcinoma and PPGL; furthermore, immunotherapies targeting PD-L1 (programmed death-ligand 1) checkpoint protein (e.g., pembrolizumab, ipilimumab, nivolumab) are currently under clinical investigation [37,38,39,40,41]. Poly ADP-ribose polymerase (PARP) inhibitors (e.g., olaparib) represent another perspective in patients harboring *SDHx* mutations due to elevated levels of succinate and NAD^+^ inhibiting homologous recombination-based DNA repair mechanism which is known to be corrected by PARP, thus keeping aberrant cells alive [42]. Furthermore, there are two kinase signaling pathways (PI3K-Akt-mTOR and Ras-Raf-Erk) affected by mutations of *RET*, *MAX*, *NF1*, and *TMEM127*, which can be inhibited by kinase signaling inhibitors (e.g., the mTOR inhibitor everolimus) [43]. Isotope therapies such as ^131^I-MIBG or somatostatin-analogue-based radiotherapies are also effective [32]. For more details on the current trials in PPGL, the reader is referred to the article by Ilanchezian et al., 2020 [44].

Given the difficulties in PPGL diagnosis, especially the lack of markers of malignancy, non-coding RNA (ncRNA) molecules are gaining increasing attention, as they have been proven to be useful in other neoplasms, as well [45].

## 2. Classification of ncRNA 

Recent progress in the field of molecular biology has revealed that only 1–2% of the transcripts encode for protein (mRNA: messenger RNA), while 90% of the genomic DNA is transcribed. Most of these are transcribed as non-coding RNA; nevertheless, ncRNAs still bear major biological functions [46]. They are epigenetic modulators of gene expression by chromatin remodeling, transcriptional regulation, and posttranscriptional modification. ncRNAs can further be classified as structural ncRNAs, like ribosomal RNAs (rRNAs), transfer RNAs (tRNAs), small nuclear RNAs (snRNAs), small nucleolar RNAs (snoRNAs), and as regulatory ncRNAs, including microRNAs (miRNAs, miRs), PiWi-interacting RNAs (piRNAs), small interfering RNAs (siRNAs), long non-coding RNAs (lncRNA), enhancer RNAs (eRNAs), and circular RNAs (circRNAs) [47,48]. These molecules span across the landscape of cancer biology. Tumors are inherently genetic diseases that derange cellular homeostasis and work towards cellular growth. Non-coding RNA molecules have been shown to be implicated in the pathogenesis of tumors [49,50].

**Figure 2:**
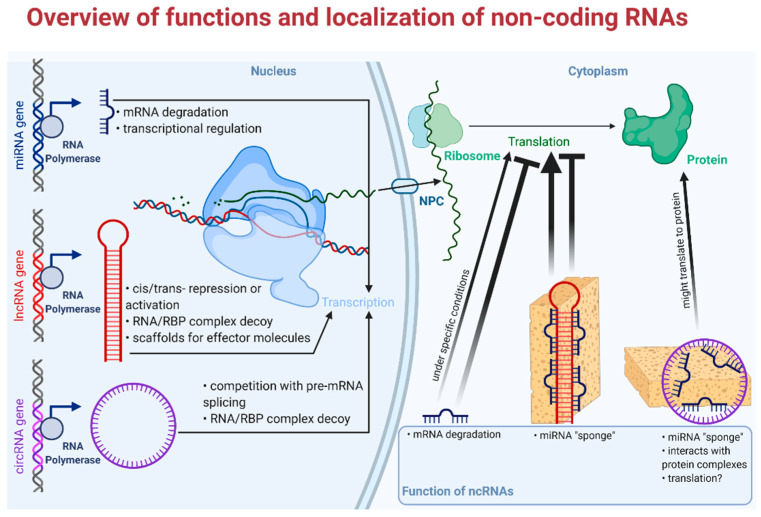
Overview of functions and localization of non-coding RNAs. RBP: RNA binding protein; NPC: nuclear pore complex. Faded arrowhead lines indicate activation; faded blunt-head lines indicate inhibition.

Long non-coding RNAs (usually from 200 to thousands of nucleotides long) are evolutionarily conserved and highly specific to cell/tissue types [51]. lncRNAs have been recently shown to be implicated in important regulatory mechanisms, as it was a long standing view not only about lncRNAs, but also about circRNAs to add no further values than being byproducts of their cognate mRNAs [52]. Surprisingly, the number of lncRNA coding genes even exceeds the number of protein coding genes, but the function of the bulk of them remains to be identified. Cellular mechanisms of lncRNAs relate to their localization within the cell. For example, nuclear transcripts control chromatin functions, transcription, and RNA processing; on the other hand, cytoplasmic lncRNAs have an effect on mRNA stability, translation, and cellular signaling (Figure 2). In different circumstances, functions of lncRNAs not only involve intracellular mechanisms, but may also act on an intercellular level, e.g., contribute to development of the tumor microenvironment and other hallmarks of cancer [53]. 

The relevance of circular RNAs (covalently bonded 3′ and 5′ ends) in biological and pathological processes has been shown only recently [54]. These peculiarly stable, evolutionarily conserved molecules play major roles mainly in the post-transcriptional regulation of gene expression e.g., by acting upon transcriptional, translational machinery or by sponging microRNAs (Figure 2). Furthermore, altered expression of circRNAs has been described in various tumors; for example, *circHIPK* functions as a miRNA sponge in colorectal, hepatocellular, kidney, prostate, breast, gastric, and bladder cancer, while *hsa_circ_0004277* seems to be a potential biomarker and therapeutic target in acute myelogenous leukemia [55,56]. CircRNAs are formed from the intron-containing pre-mRNA in a process called “backsplicing”, but they are expressed in a different manner to their linear counterparts. Differential expression of circRNAs is explicable via, e.g., different structures of introns (reverse complementary repeat sequences) [57]. Furthermore, one of the most interesting aspect of circRNAs is their potential as biomarkers, as they exhibit high stability compared to other linear RNAs and they show cell-type-specific expression profiles [58,59]. There are four different types of circular RNAs: i. 2′-5′ intronic circRNA (ciRNA) localized in the nucleus, ii. 3′-5′ exon-intron circRNA (EIciRNA) also with nuclear localization, iii. intergenic circRNA located in the cytoplasm, and the most abundant, iv. exonic circRNA (ecircRNA), also localized in the cytoplasm [60,61]. Circular RNAs exert their biological potential via two mechanisms: via backsplicing and subsequent competition with their linear counterpart from the host gene and via trans-regulatory effect of the circRNA end product. Their effect on gene expression can further be divided into six mechanisms: i. sequestration of miRNA, so-called miRNA “sponges”; ii. stimulation of initiation and elongation of transcription by acting upon RNA polymerase II; iii. down-regulation of cognate mRNAs by attenuation of linear splicing; iv. through protein binding they are able to inhibit translational activity; v. a portion of them is protein coding circRNA; vi. circRNAs can alter enzymatic reactions by forming ternary complexes [62,63]. 

MicroRNAs (miR, miRNA) have been proposed to have a major impact on biological function of tumors and are of great interest as candidates of liquid biopsy. Mature miRs are single-stranded, short RNA molecules comprising 19–25 nucleotides, that are also evolutionarily conserved and encoded by proper miRNA genes [64]. They have a role in the regulation of 30–60% of human genes in epigenetic, posttranscriptional modification, without altering the very sequence of DNA. MicroRNAs are shown to behave similarly to transcription factors (TF). While TFs exert their activating or silencing effect by binding to a specific region of the promoter in the nucleus, miRNAs bind to the 3′ UTR (*untranslated region*) of their mRNA target, hence degrading them or blocking their translation in the cytoplasm; however, they can also act in the nucleus (Figure 2) [65,66]. Today, we see an abundance of the biological functions of miRs. Their pleiotropic effects include the regulation of cell cycle and differentiation, cell proliferation, hormone secretion, apoptosis and are also implicated in the regulation of hemopoiesis, immune functioning, and ontogenesis. Several pathogenic processes including tumorigenesis, autoimmune disorders, and vascular diseases among others can be found to be associated with altered miRNA expression [67]. Another important aspect of miRs is their cell- and tissue-specific expression. Cell-specificity means that the expression of miR is different in various tissues, moreover a certain miR can act differently, either as a silencer or rarely an activator in different tissues [65]. In line with this, a miR can be a tumor suppressor in one tissue and an oncogene in another making regulation via miR rather complex and local. Thanks to their abundance and exceptionally high stability, miR expression profiles can be studied in easily accessible archived formalin-fixed paraffin-embedded tissue samples and—being secreted—even in bodily fluids [68,69]. These aforementioned features make microRNAs some of the most studied molecules in the field of minimally invasive diagnostics of neoplastic and non-neoplastic diseases—especially true with “hard-to-diagnose” entities like adrenal tumors or thyroid tumors [70].

## 3. Non-Coding RNAs in PPGL

### 3.1. CircRNAs in PPGL

To date, only one study has investigated the expression pattern of circular RNAs in PPGL, suggesting its role in histone methylation [71]. The authors performed RNA sequencing on circRNA transcripts of tumor tissue compared to adjacent normal tissue from PPGL patients. In the discovery cohort, seven patients were randomly assigned in order to perform transcriptome analysis, which revealed 3927 mRNAs, 283 miRNAs, and 367 circRNAs to be differentially expressed. The top 11 differentially expressed circRNAs have been validated by real-time quantitative PCR (RT-qPCR) on 33 pairs of PPGL tumor tissues and adjacent normal tissues from snap-frozen samples. Out of 367 differentially expressed circRNAs 112 were shown to be down-regulated and 255 were up-regulated. The top three overexpressed histone methylation-related circRNAs (*hsa_circ_0000567*, *hsa_circ_0002897*, and *hsa_circ_0004473*) related to histone methylation were identified and validated as well as their miRNA targets (Table 1). These three circRNAs were also found to be differentially expressed in the peripheral blood from 16 PPGL patients and 16 healthy individuals. By bioinformatical analysis, *hsa_circ_0000567* was predicted to bind *hsa-miR-96-3p*, which is involved in the regulation of histone methylation [71]. Furthermore, a coding-non-coding gene co-expression network (CNC) was established by mapping of circRNA-miRNA-mRNA transcripts involving known PPGL susceptibility genes. It has been proposed that these circRNAs related to histone methylation function as miRNA sponges. 

Limitations of this study include the small number of patients included and that the control samples were derived from normal tissues adjacent to the tumor, instead of from individuals adrenalectomized for other (non-PPGL-related) causes. Epigenetic alterations can precede tumor formation (hence the prognostic value) and play major role in cell-to-cell communication (hence the therapeutic value) and by analyzing differential expression profiles, protein-protein interactions, gene set enrichment, dimensionality reduction, and tissue composition, it was elucidated that normal tissues adjacent to the tumor represent a unique in-between state concerning the molecular landscape [80]. Pan-cancer proinflammatory reaction in the adjacent endothelium was also suggested to bias the outcome of the normal tissue adjacent to the tumor as control tissue. Moreover, in this study, pathway analyses were also restricted only to bioinformatical predictions and the physical interaction between *hsa_circ_0000567* and *hsa-miR-96-3p* has not been confirmed, either. 

### 3.2. Long Non-Coding RNAs in PPGL

It is important not only to detect the expression profiles of non-coding RNAs, but also to have an understanding of their mechanistic interaction with other regulatory molecules. For example, some lncRNAs have binding sites with microRNAs, thus sequestering them, thereby increasing the expression of their target genes. 

In a competing endogenous RNAs (ceRNA) bioinformatics study, the expression of mRNAs, miRNAs, and lncRNAs in PPGL related to non-tumorous tissues were analyzed in datasets downloaded from the Cancer Genome Atlas (TCGA) [76]. To design a ceRNA study, it is a basic principle that the more binding sites the lncRNA have, the stronger they can down-regulate miRNA, thus inhibiting mRNA degradation. The authors observed 554 lncRNAs, 1775 mRNAs, and 40 miRNAs to be differentially expressed, from which 23 lncRNAs, 22 mRNAs, and 6 miRNAs were selected to build the ceRNA network. Twenty-three lncRNAs were identified to be differentially expressed in PPGL, and among them two were related to overall survival, i.e., lncRNA *BSN-AS2* and *C9orf147*, without having been described previously as related to other diseases. LncRNA *BSN-AS2* and *C9orf147* are future candidates to investigate their roles in tumorigenesis as their overexpression was associated with poor prognosis; moreover, the underexpression of *C9orf147* was associated with good prognosis (Table 1). Up-regulation of *BSN-AS2* has been observed in 183 pheochromocytoma patients related to a very low number (3) of control samples. As reported by the study, *BSN-AS2* might exert its impact on prognosis through altering receptor-type tyrosine-protein phosphatase eta (*PTPRJ*) mRNA expression by interacting with *miR-195* based on bioinformatical predictions. *PTPRJ* underexpression was found to be correlated with good prognosis. On the other hand, *BSN-AS2* competes with *miR-193b*, *miR-195* and *miR-497*, thereby modulating *TGFBR3* mRNA, which was positively associated with OS. Interestingly enough, *TGFBR3* mRNA levels were found to be underexpressed in pheochromocytoma patients, therefore, we are still in need of explanation of divergent expression levels between *TGFBR3* mRNA and *BSN-AS2* lncRNA. The findings of this bioinformatics study also need to be validated experimentally.

A recently published study about the transcriptome analysis of lncRNAs in PPGL revealed lncRNA phenotypes that can distinguish PPGL subtypes [81]. In the *SDHx* subtype, a putative lncRNA *BC063866* was found to be able to distinguish between metastatic tumors and tumors that remain indolent. lncRNA *BC063866* was found to be related to some of the genes involved in metastatic signature of various tumors such as *CDH19, ERBB3*, *PLP1*, and *SOX10*. Interestingly, these genes are also involved in neural crest and glial development [82]. Furthermore, lncRNA *BC063866* was found to be an independent risk factor for poor outcome in *SDHx* mutants, although this marker should be replicated in large prospective cohorts, as well. 

Additionally, in a more recent ceRNA bioinformatics study, the previously described *miR-195-5p* and *miR-34a-5p* were predicted to be involved in the following two lncRNA–miRNA–mRNA axes: *AP001486.2/hsa-miR-195-5p/RCAN3* and *AP006333.2/hsa-miR-34a-5p/PTPRJ* respectively, functioning as tumor suppressors [83]. Higher expression levels of *RCAN3* (regulator of calcineurin 3) and *PTPRJ* in PPGL compared with normal adjacent tissue were experimentally validated by immunohistochemistry analysis. Matching with normal adjacent tissue might bias the results, as it was outlined before. The ceRNA study also revealed *RCAN3* as a good prognostic marker. In contrast to the previous study [76], this bioinformatical approach revealed underexpressed PTPRJ to be related to unfavorable prognosis. The controversial results concerning the relevance of *PTPRJ* highlight the limitations of bioinformatical analyses and the need for focused translational studies to establish the marker potential of a given coding or non-coding RNA molecule. *PTPRJ* might be involved in malignancies at different levels acting both as a tumor suppressor, but also in the regulation of antitumoral T-cell activity [84,85]. In a similar manner, *RCAN3* is implicated in the calcineurin–nuclear factor of activated T cells (NFAT) pathway-mediated immune response and also acts as a tumor suppressor [86]. It is also noteworthy that *miR-483-5p*, *miR-195*, and *miR-34a* were shown to be differentially expressed in adrenocortical cancer, as well [79,87].

### 3.3. MicroRNA in PPGL

According to one of the first studies from our research group on the miRNA expression profiles in FFPE samples of PPGL of various genetic backgrounds, *miR-139-3p*, *miR-541* and *miR-765* in VHL showed significantly higher expression compared to sporadic benign pheochromocytomas [68]. Altered expression of *miR-139-3p* has been demonstrated in various types of cancer [88,89,90]. *miR-541* has been shown to be upregulated in VHL compared with sporadic recurring pheochromocytomas (Table 1). Another finding has been the overexpression of *miR-885-5p* in MEN2-related pheochromocytoma compared with VHL- NF1-, sporadic recurring, and sporadic benign pheochromocytomas. Upregulated expression of *miR-1225-3p* has been found in sporadic recurrent pheochromocytomas in comparison to benign pheochromocytomas that raised its potential as a marker of PPGL recurrence. By using a bioinformatics pathway analysis approach, we raised the relevance of Notch-signaling in pheochromocytoma recurrence, and there are in vitro data showing the anti-proliferative potential of Notch-modulation in pheochromocytoma [91].

The previously detailed ceRNA network study in pheochromocytoma revealed the up-regulation of *miR-137* and *miR-375* and down-regulation of *miR-193b*, *miR-195*, *miR-497*, and *miR-508* [76]. 

The aforementioned recent ceRNA study also shed light on *miR-148b-3p* and *miR-338-3p* in respect of favorable prognosis and overall survival in PPGL [83].

Studies aimed at understanding miR expression pattern changes between benign and metastatic PPGL are pivotal in order to be able to differentiate between these two entities. Whole-genome microarray profiling revealed eight miRNAs to be differentially expressed [74]. In this study, “malignancy” was established when there was clinical evidence of tumor from “extra-chromaffin” sites corresponding to the current WHO definition of metastatic PPGL, but also when there was extensive local invasion. Significantly altered expression of *miR-101*, *miR-183*, and *miR-483-5p* was revealed in metastatic pheochromocytoma tissues versus benign ones and validated by RT-qPCR. Among them, *miR-101* and *miR-183* significantly differed in *SDHB* mutant vs. wild type samples and interestingly, *miR-483-5p* had significantly lower expression in *SDHB* mutant malignant pheochromocytoma compared to all other malignant pheochromocytomas. Furthermore, *miR-101*, *miR-183*, and *miR-483-5p* were measurable from serum samples, as well. In practice, this might raise the possibility that a patient without *SDHB* mutation might be screened for miR expression profile changes to assess the risk of malignancy. In another study investigating snap-frozen samples, significantly higher expression of *miR-483-5p* in metastatic PPGL was found, as well, validated by RT-qPCR [72]. The definition of metastatic disease corresponded to the WHO definition in this study, i.e., only tumors with metastases at “extra-chromaffin” sites were considered metastatic. On the other hand, lower expression of the general tumor suppressor miRNAs *miR-15a* and *miR-16* were revealed in metastatic versus benign tumors. *miR-15* and *miR-16* were raised as potential therapeutic targets, as their restoration in expression promoted cell death, partly through the down-regulation of *CCND1* (Cyclin D1) in metastatic rat pheochromocytoma cells [72]. Up-regulation of *miR-483-5p* in metastatic tumors corresponded to the amplification of *IGF2* (insulin-like growth factor 2) mRNA due to their co-expression from the same locus [72]. *IGF2* protein and mRNA were shown to be significantly increased in metastatic PPGL, which is consistent with other studies investigating the relationship between *IGF2*, *miR-483-5p*, and adrenocortical carcinoma, where *miR-483-5p* is also overexpressed in comparison to benign adrenocortical adenomas [70,79]. Moreover, *miR-483-5p* is a marker of worse disease-free survival in metastatic pheochromocytoma [72]. 

As mentioned before, *miR-210* (a general hypoxamiR [92]) is a key molecule in pseudohypoxia-type PPGL functioning as a master regulator [77]. When PPGL was compared with normal adrenal medullary tissues, overexpressed *miR-210* was significantly associated with *SDHx* or *VHL* mutant genotypes known to exhibit the pseudohypoxia phenotype [78]. 

The aforementioned *miR-96* and *miR-183* were described to contribute to the differentiation block of cells of *SDHB* mutated tumors [93]. An integrative study of expression signatures of PPGL revealed that *miR-382* targeting *SOD2* (superoxide dismutase 2) and *C-MYC* was up-regulated in tumors of most genetic backgrounds (*VHL*, *SDHB*, *SDHD*, *RET*) except in MAX mutants [75]. Up-regulation of *miR-137* was also observed in most genetic backgrounds (*VHL*, *SDHB*, *SDHD*, *RET*) except in MAX. *miR-137* possibly down-regulates *RUNX2*, *KDM5B* (histone H3 Lys4 demethylase) and interferes with *IDH1–EGLN* pathway, thus regulating neuronal gene activity as it has been previously reported [94]. *miR-885-5p* (interestingly a tumor suppressor) and *miR-488* were specific to *MEN2-related* PPGLs. *miR-133b* was related to *VHL*-type PPGLs. Robust upregulation was identified with *miR-96* especially in *SDHB* mutants [75].

**Figure 3:**
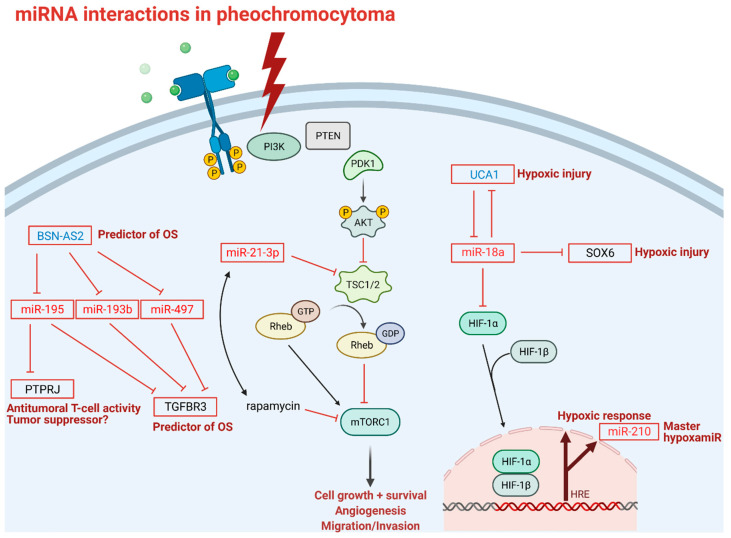
miRNA interactions in pheochromocytoma: Transmembrane tyrosine kinase receptor activation is the first step in the mTOR signaling pathway; thunderbolt represents activation of mTOR pathway in pheochromocytoma; P indicates phosphorylation sites; blunt-head lines indicate inhibition; faded arrows indicate downstream activation; solid arrows indicate direct activation; right-angle arrow indicates gene expression. Abbreviations: BSN-AS2: long non-coding RNA BSN-AS2; OS: overall survival; PTPRJ: receptor-type tyrosine-protein phosphatase eta; TGFBR3: transforming growth factor beta receptor 3; PI3K: phosphoinositide 3-kinase; PTEN: phosphatase and tensin homolog; PDK1: phosphoinositide-dependent kinase 1; AKT1: a serine/threonine protein kinase; TSC1/2: tuberous sclerosis complex subunit 1; Rheb: Ras homolog enriched in brain; GTP: guanosine triphosphate; GDP: guanosine diphosphate; mTROC1: mammalian target of rapamycin complex 1; rapamycin: mTOR inhibitor; UCA1: long non-coding RNA urothelial cancer associated 1; SOX6: SRY (sex determining region Y)-box 6; HIF-1 α/β: hypoxia inducible factor 1 subunit α/β; HRE: hypoxia response element; PC-12: pheochromocytoma 12 cell line, OS: overall survival. Note that miR-18 mediated down-regulation of HIF-1α has only been established in lung cancer stem-like cells, choroidal endothelial cells, and in breast cancer xenograft model and not yet in pheochromocytoma cells.

In neuronal pheochromocytoma 12 cells (PC-12) *miR-18a* is involved in hypoxic responses through down-regulation of lncRNA urothelial carcinoma associated 1 (UCA1), sex determining region Y-box 6 (SOX6), and hypoxia inducible factor 1 subunit α (HIF-1α) [95]. However, the regulatory functions of *miR-18a* on *HIF-1α* have only been described previously in lung cancer stem-like cells, choroidal endothelial cells, and in a breast cancer xenograft model [96,97,98]. Given the tissue-specific nature of miRNA expression and action, the interaction between *miR-18a* and HIF-1α in PPGL should be investigated in pheochromocytoma cells. Under hypoxic conditions, UCA1 is upregulated, making cells more prone to hypoxic injuries through the putative down-regulation of *miR-18a*. Down-regulation of UCA1 is associated with the attenuation of hypoxic injuries. Furthermore, UCA1 directly targets and down-regulates *miR-18a* and vice versa, and the up-regulation of *miR-18a* alleviates hypoxic injury through downregulation of UCA1. Similar to UCA1, SOX6 also acts as a provoking factor in hypoxic injuries and inhibition of SOX6 leads to an ease of hypoxic injury (Figure 3).

MiR profiling also holds therapy-modifying potential in precision medicine. A recent study revealed a new regulatory axis of *miR-21-3p*/TSC2/mTOR signaling pathway as a future target for treatment, as *miR-21-3p* showed significant association with sensitivity to rapamycin, thus, *miR-21-3p* could be a marker for mTOR inhibitor therapy (Figure 3) [73]. This study not only shed light on miR profiling as a tool in risk stratification in PPGL, but also gives us a predictive biomarker accessible via liquid biopsy to investigate in a larger cohort in the future. 

It is quite intriguing that some microRNAs seem to be differentially expressed between both benign and metastatic PPGL and benign and malignant adrenocortical tumors. These include *miR-483-5p*, *miR-195*, and *miR-34a* [72,74,76,79,83,87]. As the adrenal cortex is of mesodermic origin, whereas the adrenal medulla is of ectodermic origin, these common changes in microRNA expression might even suggest some common adrenal-specific features in tumorigenesis. Confirmation in larger cohorts is warranted. 

Based on these significant differences in expression profiles, miR, lncRNA, and circRNA profile analysis are still one of the chief candidates for an adjunct diagnostic marker for “hard-to-diagnose” tumors. 

### 3.4. ncRNAs as Therapeutic Targets in PPGL

Currently, there are no clinical studies evaluating ncRNAs as therapeutic targets in PPGL. Since treatment options for metastatic PPGL are rather limited, novel molecular targets are intensively sought for. We can only hypothesize on the relevance of ncRNAs in the treatment of PPGL from specific observations. Some of the ncRNA detailed above might represent potential treatment targets or exploited as markers of therapy-modifying potential. For example, miR-21-3p was shown to be correlated with rapamycin sensitivity, thus, miR-21-3p could be a marker for mTOR inhibitor therapy in PPGL (Figure 3) [73]. Detailed preclinical molecular investigations will be necessary to define the ncRNA that could be exploited as treatment targets (e.g., restoration of underexpressed “tumor suppressor” ncRNA expression or targeting overexpressed oncogenic ncRNA by small interfering RNA), but there would be quite a long way ahead before the clinical application of any treatments targeting these pathways given the numerous difficulties in such treatment strategies (e.g., problems of administration, question of the vector, off-site effects, etc.) [99].

## 4. Conclusions

Pheochromocytoma was originally named after its microscopic and staining features and due to the complex nature of the disease, current diagnostics encompasses not only imaging and laboratory tests, but also the quest for new biomarkers on the horizon of an ever-evolving field of non-protein-coding ribonucleic acids. The emerging role of non-coding RNA in the setting of clinical evaluation and therapeutic approaches of clinically challenging tumors is an attractive candidate for precision medicine. By studying non-coding RNA, we might be able to double attack the therapeutic and the diagnostic ends of PPGL in our efforts towards making a reliable tool for the distinction and targeted therapy of metastatic and benign tumors.

## Figures and Tables

**Table 1:** Functions of ncRNAs with altered expression in PPGL.

ncRNA	Method and Sample (Number of Patients)	Expression Alteration and Suggested Role	Ref.
*hsa-circ-0000567*	RNA-seq (M = 7, N = 7)/ RT-qPCR (M = 33, N = 33)	related to histone methylation; predicted to bind *hsa-miR-96-3p*	[71]
*hsa-circ-0002897*	RNA-seq (M = 7, N = 7)/ RT-qPCR (M = 33, N = 33)	related to histone methylation	[71]
*hsa-circ-0004473*	RNA-seq (M = 7, N = 7)/ RT-qPCR (M = 33, N = 33)	related to histone methylation	[71]
*hsa-miR-15a*	Microarray (M = 12, B = 12, N = 5)/ RT-qPCR (B = 10, M = 10)	tumor suppressor; promotes cell death via downregulation of *CCND1*; underexpressed in metastatic pheochromocytoma	[72]
*hsa-miR-16*	Microarray (M = 12, B = 12, N = 5)/ RT-qPCR (B = 10, M = 10)	tumor suppressor; promotes cell death via downregulation of *CCND1*; underexpressed in metastatic pheochromocytoma	[72]
*hsa-miR-21-3p*	Discovery cohort: 443 metastatic vs. non-metastatic samples; Validation cohort: 49 non-metastatic and 8 non-metastatic vs. metastatic	regulates TSC2/mTOR axis; association in expression with sensitivity to rapamycin	[73]
*hsa-miR-96-3p*	RNA-seq (M = 7, N = 7)/ RT-qPCR (M = 33, N = 33)	regulates histone methylation; predicted to bind *hsa-circ-0000567*	[64]
*hsa-miR-101*	Microarray (M = 8, B = 42, N = 21)/ RT-qPCR (M = 25, B = 36, N = 21)	overexpression in *SDHB* mutant; overexpression in metastatic pheochromocytoma	[74]
*hsa-miR-133b*	Microarray (M = 5, B = 58, N = 6)/ RT-qPCR (M/B = 28, N = 2)	overexpression in VHL type PPGLs	[75]
*hsa-miR-137*	Microarray (M = 5, B = 58, N = 6)/ RT-qPCR (M/B = 28, N = 2)	overexpression in PPGL; downregulates *RUNX2*, *KDM5B*, *IDH1*	[75]
*hsa-miR-139-3p*	Microarray (M/B = 24)/ RT-qPCR (M/B = 33)	overexpression in VHL pheochromocytoma	[68]
*hsa-miR-183*	Microarray (M = 8, B = 42, N = 21)/ RT-qPCR (M = 25, B = 36, N = 21)	overexpression in *SDHB* mutant; overexpression in metastatic pheochromocytoma	[74]
*hsa-miR-193b*	RNA-seq (B/M = 183, N = 3)	underexpression in PPGL; mediates *TGFBR3* expression through *BSN-AS2* competition	[76]
*hsa-miR-195*	RNA-seq (B/M = 183, N = 3)	underexpression in PPGL; mediates *TGFBR3* expression through *BSN-AS2* competition	[76]
*hsa-miR-210*	RT-qPCR (B/M = 39)	overexpression in pseudohypoxia subtype; tumor hypoxia marker; associated with *SDHx* or *VHL* mutations	[15,77,78]
*hsa-miR-375*	RNA-seq (B/M = 183, N = 3)	overexpression is PPGL	[76]
*hsa-miR-382*	Microarray (M = 5, B = 58, N = 6)/ RT-qPCR (M/B = 28, N = 2)	overexpression in tumors with *VHL*, *SDHB*, *SDHD*, *RET* mutations; targeting *SOD2*, *C-MYC*	[75]
*hsa-miR-483-5p*	Microarray (M = 12, B = 12, N = 5)/ RT-qPCR (B = 10, M = 10)	overexpression in metastatic PPGL; underexpression in *SDHB* among metastatic PPGL; worse disease-free survival in metastatic PPGL; co-amplification with *IGF2* in metastatic adrenal tumors	[72,74,79]
*hsa-miR-488*	Microarray (M = 5, B = 58, N = 6)/ RT-qPCR (M/B = 28, N = 2)	overexpression in *RET* PPGL	[75]
*hsa-miR-497*	RNA-seq (B/M = 183, N = 3)	underexpression in PPGL; mediates *TGFBR3* expression through *BSN-AS2* competition	[76]
*hsa-miR-508*	RNA-seq (B/M = 183, N = 3)	underexpression in PPGL	[76]
*hsa-miR-541*	Microarray (M/B = 24)/ RT-qPCR (M/B = 33)	overexpression in VHL pheochromocytoma	[68]
*hsa-miR-765*	Microarray (M/B = 24)/ RT-qPCR (M/B = 33)	overexpression in VHL pheochromocytoma	[68]
*hsa-miR-885-5p*	Microarray (M/B = 24)/ RT-qPCR (M/B = 33)	overexpression in MEN2 PPGL	[68]
*hsa-miR-1225-3p*	Microarray (M/B = 24)/ RT-qPCR (M/B = 33)	overexpression in sporadic recurrent PPGL	[68]
*lncRNA BSN-AS2*	RNA-seq (B/M = 183, N = 3)	negative association with OS; mediate TGFBR3 expression through *miR-193b*, *miR-195*, *miR-497* competition	[76]
*lncRNA C9orf147*	RNA-seq (B/M = 183, N = 3)	positive association with OS	[76]

B—benign; M—metastatic; N—normal/control; OS—overall survival.
